# Insights into KMT2A rearrangements in acute myeloid leukemia: from molecular characteristics to targeted therapies

**DOI:** 10.1186/s40364-025-00786-y

**Published:** 2025-05-13

**Authors:** Sara Zehtabcheh, Hamed Soleimani Samarkhazan, Marjan Asadi, Mitra Zabihi, Sahar Parkhideh, Mohammad Hossein Mohammadi

**Affiliations:** 1 Student Research Committee, Department of Hematology and Blood Banking, School of Allied Medical Sciences, Shahid Beheshti University of Medical Sciences, Tehran, Iran; 2 Department of Hematology and Blood Banking, School of Allied Medical Sciences, Shahid Beheshti University of Medical Sciences, Tehran, Iran; 3 Hematopoetic Stem Cell Research Center, Shahid Beheshti University of Medical Sciences, Tehran, Iran

**Keywords:** Acute myeloid leukemia, KMT2A rearrangement, Menin inhibitors, DOT1L inhibitors

## Abstract

Acute myeloid leukemia (AML) with KMT2A rearrangements (KMT2A-r) represents a highly aggressive and prognostically unfavorable subtype of leukemia, often resistant to standard treatments and associated with high relapse rates. KMT2A-r, found in 3–10% of adult AML cases, disrupt epigenetic regulation by forming chimeric proteins that activate oncogenic pathways like HOXA and MEIS1. These fusion proteins recruit cofactors such as Menin and DOT1L, driving leukemogenesis through abnormal histone methylation. Diagnosing KMT2A-r AML requires precision, with traditional methods like FISH and RT-PCR being complemented by advanced technologies such as next-generation sequencing (NGS) and machine learning (ML). ML models, leveraging transcriptomic data, can predict KMT2A-r and identify biomarkers like LAMP5 and SKIDA1, improving risk stratification. Therapeutically, there is a shift from chemotherapy to targeted therapies. Menin inhibitors (e.g., Revumenib, Ziftomenib) disrupt the Menin-KMT2A interaction, suppressing HOXA/MEIS1 and promoting differentiation. DOT1L inhibitors (e.g., Pinometostat) show promise in combination therapies, while novel approaches like WDR5 inhibitors and PROTAC-mediated degradation are expanding treatment options. Despite progress, challenges remain, including optimizing minimal residual disease monitoring, overcoming resistance, and validating biomarkers. This review emphasizes the imperative to translate molecular insights into personalized therapeutic regimens, offering renewed hope for patients afflicted by this historically refractory malignancy.

## Introduction

Acute myeloid leukemia (AML) is a heterogeneous hematologic malignancy characterized by clonal expansion, uncontrolled proliferation, and differentiation arrest of myeloid progenitor cells. Among its diverse subtypes, AML with KMT2A rearrangements (KMT2A-r) is recognized as a clinically and biologically distinct entity in the world health organization (WHO) and the 2022 international consensus classification (ICC) [1–3]. KMT2A, previously known as mixed-lineage leukemia (MLL), plays a critical role in hematopoiesis, and its rearrangements implicated in approximately 3–6% of adult de novo AML and up to 10% of therapy-related AML cases [4]. These rearrangements involve in-frame fusions with over 100 partners; the most common KMT2A-r cases found in adult AML are t(9;11) KMT2A-MLLT3, t(6;11) KMT2A-MLLT4, t(11;19) KMT2A-ELL, and in-frame partial tandem duplications (PTD) (Table 1). The european leukemia net (ELN) risk stratification categorizes KMT2A-r AML into favorable, intermediate, and poor-risk groups based on cytogenetic complexity and clinical factors [5, 6]. However, regardless of fusion partners, KMT2A-rearranged leukemias typically exhibit poor prognoses, characterized by progression-free survival (PFS) rates of 30–40% and overall survival (OS) rates < 25% [7]. The disease generally presents with a rapid onset, aggressive progression, and a significantly worse prognosis compared to non-KMT2A-rearranged AML patients. Complex karyotypes, characterized by multiple chromosomal aberrations, are hallmarks of KMT2A-r AML and correlate with aggressive clinical behavior.

The recognition of KMT2A-r as a primary driver in AML has spurred efforts to develop targeted therapies that counteract the oncogenic effects of these fusion proteins. Recent advancements in molecular biology have improved our understanding of how KMT2A fusion proteins dysregulate gene expression, promoting leukemogenesis and treatment resistance [8]. The KMT2A-r AML is characterized by elevated HOXA9/MEIS1 gene expression [8]. These oncogenic proteins interact with cofactors such as DOT1L and Menin, forming the basis for emerging targeted therapies. In recent years, the development of targeted agents specifically designed to inhibit KMT2A fusion proteins has demonstrated high specificity and efficacy against malignant cells [9]. Recent advancements include the FDA-approved Menin inhibitor revumenib, and innovative approaches targeting KMT2A complexes via PROTACs, WDR5 inhibitors [10]. Understanding the molecular underpinnings of KMT2A-r AML is essential for improving prognosis and tailoring personalized treatment strategies [11, 12]. This review aims to provide a comprehensive overview of the molecular characteristics, prognostic implications, and emerging targeted therapies for KMT2A-rearranged AML, highlighting recent advances that lead the way toward precision medicine in this high-risk leukemia subtype.

## Characteristics of KMT2A

### Structure and function of KMT2A

The KMT2A gene, located on chromosome 11q23, encodes a nuclear protein critical for epigenetic regulation. KMT2A is a member of the Trithorax group (TrxG) proteins, mediating transcriptional activation by catalyzing histone H3 lysine 4 methylation (H3K4me), a hallmark of active gene promoters [13, 14]. Among the KMT2 family (KMT2A–KMT2G), KMT2A is uniquely implicated in hematologic malignancies due to its essential role in hematopoietic stem cell (HSC) differentiation and its propensity for chromosomal rearrangements [15, 16].

**Fig. 1:**
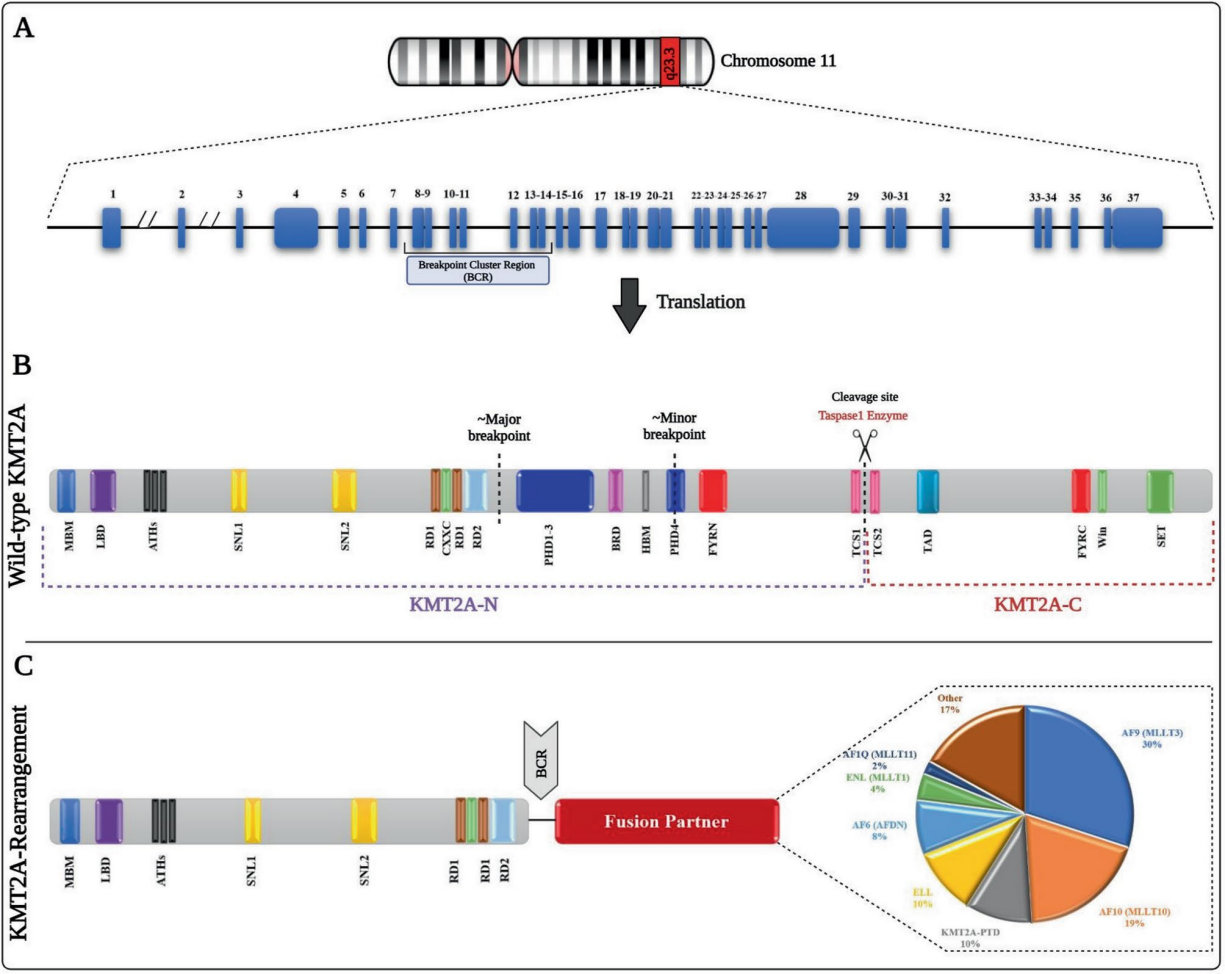
An overview of the KMT2A gene, Its structure, and rearrangements. (**A**) Gene Structure and Key Regions. The KMT2A gene on chromosome 11 band q23.3 (11q23.3) spans about 97 Kb with 37 exons coding a large nuclear protein. Its structure is organized into regions: exons 1–3 encode the Menin-binding domain (MBD) and AT-hook motifs, which contribute to DNA binding; exons 4–9 aid in transcription regulation by coding nuclear localization signals (SNL1, SNL2) and repression domains (RD1, RD2); exons 10–27 facilitate protein interaction regions, including PHD fingers (PHD1-3) and the bromodomain (BRD); and exons 28–37 are involved in histone methylation by the transactivation domain (TAD) and SET domain. A key hotspot for chromosomal breaks and translocations, the breakpoint cluster region (BCR) frequently includes major BCR, which occurs between exons 8 and 10, and minor BCR, which occurs around exon 14. It often experiences breaks leading to potential gene fusions. (**B**) post-translation processing. Once the KMT2A protein is translated, it is split into two fragments by Taspase-1. This cleavage produces the N-terminal fragment (KMT2A-N) and the C-terminal fragment (KMT2A-C). The KMT2A-C contain the SET domain, crucial for adding methyl groups to histone H3K4. (**C**) gene rearrangements and their impact. KMT2A-r occur when the gene translocate in its BCR region, fusing with various genes (such as MLLT3/AF9, MLLT10/AF10, MLLT1/ENL, or ELL). These fusions typically merge the N-terminal portion of KMT2A with segments from other genes, resulting in its C-terminal regions loss. This disruption in normal gene function is a key factor in the development of leukemia

The KMT2A protein (≈ 500 kDa) is proteolytically cleaved by taspase-1 into N-terminal (KMT2A-N) and C-terminal (KMT2A-C) fragments. KMT2A-N contains domains critical for chromatin interactions, including Menin-binding domains (MBD), AT-hook motifs (DNA binding), and repression domains (RD1/RD2), while KMT2A-C harbors the SET domain responsible for H3K4 methylation [8, 16, 17]. Post-translational processing ensures proper assembly of the KMT2A multiprotein complex, which regulates transcription elongation and histone modification (Fig. 1A, B) [18, 19].

In normal hematopoiesis, KMT2A maintains the balance between HSC self-renewal and differentiation by regulating HOX gene clusters (e.g., HOXA9, HOXA7) [20, 21]. Comprehensive studies on KMT2A function in hematopoietic development have demonstrated its essential role in definitive hematopoiesis and the proliferation of hematopoietic progenitors and stem cells in the aorta-gonad-mesonephros (AGM) region of the developing embryo. Additionally, conditional knockout studies in mice demonstrate that KMT2A loss impairs HSC proliferation and repopulation capacity, underscoring its role in definitive hematopoiesis [22, 23].

### KMT2A rearrangement

Numerous KMT2A-related genomic alterations, including chromosomal translocations, internal tandem duplications (ITDs), internal deletions, and amplifications, have been implicated in various malignancies. In hematologic malignancies, the most common structural variant of KMT2A is the KMT2A-r. In 2023, Meyer et al. analyzed 3,401 acute leukemia cases using long-distance inverse PCR (LDI-PCR) and targeted next-generation sequencing (NGS), revealing that 11q23 translocations generate in-frame KMT2A fusions with over 100 partner genes [12]. The most common KMT2A fusion partners in AML patients include AF9 (MLLT3), AF10 (MLLT10), KMT2A-PTDs, ELL, AF6 (AFDN), ENL (MLLT1), and MLLT11 (Table 1) [12]. The KMT2A fusion region typically spans 8.3 kb of breakpoint clusters, covering exons 8 through 14. KMT2A PTDs, most frequently found in exons 2 to 9, occur in approximately 10% of myelodysplastic syndrome/acute myeloid leukemia (MDS/AML) cases and are linked to disease progression and relapse [11, 24]. In most cases, chimeric KMT2A fusion proteins retain the N-terminal domains but lose the final four domains (PHD finger, bromodomain, activation domain, and SET domain). The N-terminal segment in translocation-encoded fusions loses its capacity to interact with KMT2A-C (Fig. 1C) [24].

KMT2A-r disrupt normal gene expression through dysregulated H3K4me and H3K79me, leading to altered chromatin states and activation of oncogenic pathways. KMT2A fusion proteins interact with H3K79 methyltransferase DOT1L or components of the super elongation complex through their C-terminal partners. This interaction enhances transcription elongation, sustaining activation of genes involved in anti-apoptosis and differentiation blockade [25]. Through epigenetic modulation of transcription, these interactions upregulate genes essential for hematopoietic cell proliferation and differentiation, including homebox (HOX A/B), MEIS1 (myeloid ecotropic virus insertion site 1 genes) and PBX3. Expression of HOX A/B and MEIS1 genes is elevated in immature stem cell population and decreases during hematopoietic maturation and differentiation [8, 9, 26]. This results in sustained HOXA/MEIS1 expression, blocking differentiation and driving leukemogenesis [24, 25].

**Table 1:** The most common KMT2A fusion partners in AML patients

Official Symbol	Known As	Chromosome	Exons Count	Function	Frequency in KMT2A-*r* AML Cases
MLLT3	AF9, YEATS3	9p21.3	12	Functions as a signaling hub that regulates transcription through dynamic recruitment of cofactors in normal hematopoiesis and in acute leukemia.	30%
MLLT10	AF10	10p12.31	30	A transcription factor that interacts directly with DNA, the histone complex, and other transcription factors through its domains	19%
ELL	MEN; ELL1; PPP1R68; C19orf17	19p13.11	15	Stimulating the rate of RNA polymerase II transcription by suppressing transient pausing of the polymerase at multiple sites along the DNA, essentially allowing for smoother and faster elongation during the transcription process	10%
AFDN	AF6; MLLT4; MLL-AF6; l-afadin	6q27	35	A complex modulator of cellular processes implicated in cancer progression, including signal transduction, migration, invasion, and apoptosis.	8%
MLLT1	ENL; LTG19; YEATS1	19p13.3	15	A critical component of the super-elongation complex (SEC) that regulates mRNA elongation by RNA polymerase II (RNAPII) during transcription, and hence regulates RNA expression	4%
MLLT11	AF1Q	1q21.3	2	Transcriptional regulation and chromatin remodeling.	2%

## Molecular diagnostics of KMT2A

The identification of KMT2A-r is routinely performed using screening methods that detect this alteration regardless of the specific fusion partner, owing to the extensive number of possible fusion genes. Identifying KMT2A-r is essential for enabling rapid clinical decision-making and guiding specific therapeutic approaches. Diagnostic methods for KMT2A-r include immunophenotyping using a specific antibody (NG2), split-signal fluorescence in situ hybridization (FISH), reverse transcription PCR, and NGS.

### Immunophenotyping with neuron-glial antigen-2 (NG2)

One methods for identifying KMT2A-r is immunophenotyping using an antibody targeting chondroitin sulfate proteoglycan 4 (CSPG4). CSPG4, also known as NG2, is a transmembrane proteoglycan minimally expressed in normal hematopoietic cells [27]. In contrast, NG2 is specifically expressed in approximately 90% of 11q23/KMT2A-r leukemias. A study by Smith FO et al. demonstrated that NG2 expression in blast cells was significantly correlated with FAB M5 morphology (*P* < 0.0001) and chromosomal abnormalities in 11q23 in childhood AML. The findings suggest that NG2 may be beneficial for the rapid identification of this group of individuals with poor-prognosis AML [28]. For years, NG2 has been included in diagnostic panels for the immunophenotyping of patients due to its possible predictive value for KMT2A-r in both pediatric and adult acute leukemias [29]. However, research indicates that only two specific leukemia subtypes, characterized by translocations t(4;11)(q21;q23) or t(9;11)(p13;q23), which generate the fusion genes KMT2A-AF4 and KMT2A-AF9, respectively, are uniquely associated with NG2 expression [30]. The variability of KMT2A partner genes is well-documented in AML cases, which may explain the discrepancy in diagnostic accuracy between ALL and AML samples [12, 31, 32]. Fenton et al. developed mAb 225.28, a monoclonal antibody targeting NG2, to study NG2 expression on blasts in newly diagnosed AML patients and its association with cytogenetic abnormalities and prognostic molecular markers [33]. They discovered that leukemic cells with KMT2A-r, FTL3 mutation, NPM1 mutation, and complex cytogenetic abnormalities exhibited NG2 expression. These findings suggest that NG2 expression is not specific to any AML subtype or developmental compartment, and mAb 225.28 could potentially serve as a diagnostic marker for AML [34].

### Cytogenetic analysis using FISH method

KMT2A-r can be classified into reciprocal chromosomal translocations (60.5%), insertion of 11q23 material onto a different chromosome (18.4%), 11q inversions (11.3%), 11q23 deletions (5.7%), and spliced KMT2A fusion following recombination (4.2%) [35]. Most 11q23 abnormalities can be detected through conventional cytogenetic analysis; however, this method often fails to confirm KMT2A-rs involving rare or unidentified 11q23 translocation partners or to detect cryptic KMT2A translocation. This limitation may result from complex or cryptic chromosomal abnormalities and insufficient metaphase division of leukemic cells. FISH analysis has been employed to overcome these challenges, using a commercially available KMT2A probe kit containing directly labeled KMT2A 5’ (green) and 3’ (red) probes, widely used worldwide. Occasionally, atypical signal patterns are observed, marked by an increase or loss of the 5′ green or 3′ red signals. Loss of the 3′ KMT2A signal, observed in up to 28% of KMT2A-r cases, is the most common abnormality [36]. Although FISH is effective for detecting KMT2A-r, recent studies reveal that patients with KMT2A fusions in the minor breakpoint cluster region (BCR) may show normal results when evaluated with break-apart probes [37]. This issue commonly arises in KMT2A-USP2 fusion, involving a short inversion within the 11q23 region. Consequently, the 5′ and 3′ signals of KMT2A may appear adjacent rather than distinct, mimicking a normal, non-rearranged signal profile. Studies have shown that this pattern can result in false-negative outcomes during routine FISH analysis, particularly when supplementary markers, like a 3′ KMT2A deletion, are absent to help differentiate the signal from a true normal profile. These findings underscore the limitations of FISH and emphasize the need for supplementary detection techniques in KMT2A-rearranged leukemias to enhance diagnostic accuracy.

### Molecular analysis using RT-PCR and NGS

Following initial FISH screening, single or multiplex RT-PCR is commonly employed to detect the most prevalent KMT2A fusions. Currently, DI-PCR is the most effective and validated technique for identifying both known and novel partner genes in KMT2A-r [38]. Regardless of the associated partner gene or other KMT2A abnormalities within the KMT2A BCR, LDI-PCR has proven effective in detecting rare fusions and uncovering KMT2A translocations. Advanced methodologies, including inverse PCR with isolated chromosomal patient DNA or panhandle PCR using reverse-transcribed patient RNA (cDNA), are necessary to identify novel KMT2A translocation partner genes and uncover new fusion genes. This technique enables high-throughput investigations by allowing the acquisition of genomic KMT2A fusion sequences in only four PCR cycles. This approach necessitates minimal amounts of genomic patient DNA (1 µg) and provides critical genetic information for quantitative minimal residual disease (MRD) investigations [39]. A key advantage of these DNA-based techniques is their ability to identify genomic sequences from reciprocal chromosomal fusion sites. These sequences are reliable markers for MRD studies because they are unique to each patient and only found in one type of leukemic cell.

NGS is a the most advanced molecular technology for high-throughput detection of genetic abnormalities, including KMT2A-r. NGS provides enhanced sensitivity for the detection of KMT2A-rs and facilitates the discovery of supplementary genomic changes that might influence treatment choices. Nonetheless, it continues to be costly and may exhibit limits in identifying low-expression transcripts in some cases. Recent research has validated the use of NGS methods for detecting cryptic KMT2A-r and enabling the identification of multiple genomic changes for the risk categorization of acute leukemia [40–42].

### Machine learning methodologies

Mashin learning (ML) is an innovative approach to identifying the most appropriate markers for predicting KMT2A-r in different forms of acute leukemia [43]. Lopes BA et al. utilized a ML model to identify the most appropriate markers for predicting KMT2A-r in various types of acute leukemia [43]. They found that SKIDA1 (AUC: 0.839; CI: 0.799–0.879) and LAMP5 (AUC: 0.746; CI: 0.685–0.806) were the two most important genes, irrespective of acute leukemia subtype. Overexpression of these genes had a stronger relationship with KMT2A-r [43]. LAMP5 expression effectively predicted the infrequent gene fusion KMT2A-USP2, frequently overlooked by routine methods, and the resultant gene product may serve as a viable therapeutic target in KMT2A-r leukemia. However, further research is required to validate these markers and integrate them into standard diagnostic protocols for acute leukemia [43].

Table 2 provides a comparison of KMT2A gene rearrangement diagnostic methods with respect to their advantages and disadvantages.

**Table 2:** Comparison of diagnostic methods for the KMT2A gene market

Diagnostic Method	Advantages	Disadvantages
Immunophenotyping with NG2 antibody (CSPG4)	• Rapid detection of KMT2A-r in approximately 90% of cases. • Useful for poor prognosis AML subtypes, especially FAB M5. • Potential to identify KMT2A-r in both pediatric and adult AML cases.	• Limited to specific translocations (e.g., t(4;11), t(9;11)) that produce KMT2A-AF4 and KMT2A-AF9 fusions. • Not specific to AML and may miss other KMT2A fusion genes. • Potential variability in accuracy between AML and ALL samples.
Fluorescence in situ hybridization (FISH)	• Gold standard method • Widely available and well-established technique. • Can detect cryptic KMT2A-r when combined with KMT2A probe kits. • Suitable for common KMT2A translocations.	• Limited in detecting rare or novel partner genes in KMT2A-r. • May give false-negative results in minor breakpoint regions (e.g., KMT2A-USP2 fusion). • Less effective for detecting cryptic translocations.
RT-PCR (Reverse Transcription Polymerase Chain Reaction)	• Effective for detecting known KMT2A fusions, especially in high-expression cases. • Validated for identifying KMT2A-r in both pediatric and adult populations. • Useful for identifying specific fusion genes and MRD.	• Limited to common fusion partners and not suitable for rare or cryptic fusions. • Requires fresh/frozen samples or high-quality RNA. • Not applicable for detecting novel fusion partners without specific primers.
Next-Generation Sequencing (NGS)	• High-throughput, sensitive, and can identify novel KMT2A fusion partners. • Can detect cryptic translocations and multiple genetic alterations. • Comprehensive approach for risk categorization and treatment selection.	• Costly and requires advanced bioinformatics capabilities. • May struggle with detecting low-expression transcripts or rare fusions in some cases. • High complexity and potentially overwhelming for routine clinical use.
Machine Learning (ML) Approaches	• Can predict and identify new biomarkers based on large datasets. • Offers potential for improved diagnosis and risk stratification. • Could enhance precision medicine and clinical decision-making.	• Limited annotated datasets and model interpretability. • Requires large datasets and advanced computational tools. • Still experimental, and further validation is required for clinical integration.

## Therapeutic approaches

In recent years, the development of targeted agents specifically designed to inhibit KMT2A fusion proteins has significantly advanced, leading to high specificity and efficacy against malignant cells. Targeting KMT2A fusion-interacting proteins, including DOT1L and Menin, has been proposed as a potential therapeutic strategy in preclinical studies.

### DOT1L

**Fig. 2:**
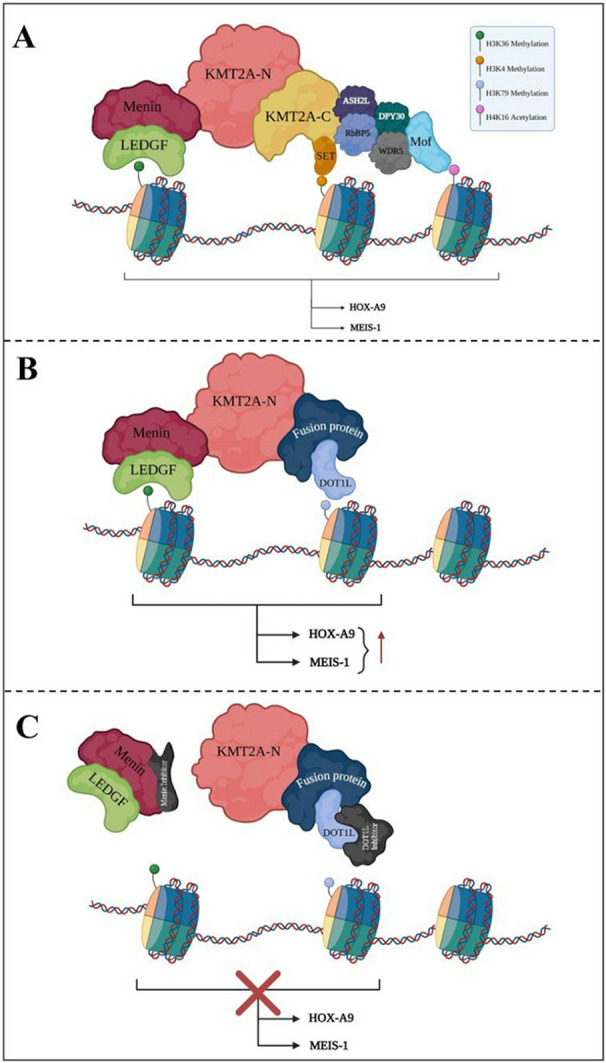
Regulation of gene expression by KMT2A and its rearrangements on HOXA9 and MEIS1 expression. (**A**) Normal KMT2A function. In a healthy cell, the KMT2A protein is split into two parts which regulate gene expression. The Menin-LEDGF complex recruits KMT2A to chromatin while the SET domain at the KMT2A-C end catalyzes H3K4 methylation, promoting gene transcription. This process turn on important genes like HOXA9 and MEIS1, which are crucial for hematopoietic differentiation and self-renewal. Other proteins, such as WDR5, ASH2L, RbBP5, DPY30, and Mof, also assist in modifying histones (as H3K36 methylation and H4K16 acetylation) to further enhance gene activity. In wild-type KMT2A, the SET domain normally regulates H3K4 methylation, which activates gene expression in a controlled manner. (**B**) Leukemogenic KMT2A rearrangements. In certain leukemias, KMT2A fuses with other genes. These fusion proteins retain the N-terminal but lose the SET domain, so they no longer add the H3K4 methylation mark. Instead, the fusion partners bring their own molecular properties, altering transcriptional regulation in leukemia. However, they still bind to chromatin via the Menin-LEDGF complex. Fusion partners recruitent of DOT1L complex leading to persistent and uncontrolled transcription of leukemogenic genes by H3K79 methylation. This causes continuous activation of HOXA9 and MEIS1, contributing to the development of leukemia. (**C**) Therapeutic approaches. New treatments are targeting these fusion proteins. Menin inhibitors disrupt its interaction with KMT2A, while DOT1L inhibitors block the aberrant H3K79 methylation. Together, they help reduce the abnormal gene expression seen in KMT2A-rearranged AML, offering a promising avenue for therapy

DOT1L is the only histone methyltransferase that specifically modifies nucleosomal histone H3 lysine 79 (H3K79) through mono-, di-, or trimethylation. It is located on chromosome 19p13.3 and comprising 25 exons [9]. DOT1L comprises approximately 1530 amino acids, featuring an N-terminal histone methyltransferase domain (amino acids 1–332) and a long C-terminal region that directly interacts with the C-terminal portion of KMT2A fusion proteins, including AF10, AF9, and eleven-nineteen lysine-rich leukemia (ENL). Unlike typical SET-domain methyltransferases, DOT1L lacks a SET domain and associates with chromatin through nucleosome interaction motifs. DOT1L is involved in various biological processes, including gene transcription, heterochromatin formation, and the DNA damage response. The interaction between KMT2A fusion partners and DOT1L facilitates transcriptional elongation and leads to abnormal recruitment of DOT1L to KMT2A target genes, including the HOXA9 cluster and MEIS1. This result in hypermethylation of H3K79 and sustained gene expression at these ectopic loci, promoting leukemic transformation (Fig. 2). Due to its essential function in KMT2A-r leukemia, DOT1L is regarded as a vital therapeutic target [44].

### DOT1L inhibitor

Pinometostat (EPZ-5676), a potent and selective small-molecule DOT1L inhibitor, blocks intracellular H3K79 methylation in a concentration- and time-dependent manner. Treatment with Pinometostat in KMT2A-rearranged cells and xenograft models reduced H3K79me2 levels, leading to decreases expression of KMT2A target genes and selective cytotoxicity toward leukemia cells [45, 46]. These findings provide strong evidence supporting DOT1L inhibition as a foundation for targeted therapies against KMT2A-r leukemia currently under clinical investigation (Table 3). A phase 1 clinical trial (NCT01684150) enrolled 51 adult patients with relapsed/refractory (R/R) KMT2A-rearranged leukemia. Participants were divided into two cohorts (6 dose-escalation and 2 expansion cohorts) and received daily intravenous (IV) doses of Pinometostat at 54 and 90 mg/m² during 28-day cycles [46]. The most common adverse effects, irrespective of etiology, included fatigue (39%), nausea (39%), constipation (35%), and febrile neutropenia (35%). Although Pinometostat achieved plasma concentrations ≥ 36 mg/m² per day—levels shown in preclinical models to exert antitumor effects and inhibit DOT1L activity in patients’ leukemic blasts [45]—only two patients achieved complete remission (CR) following continuous daily IV infusion of 54 mg/m² Pinometostat. These findings suggest that DOT1L inhibition by Pinometostat monotherapy is insufficient to produce meaningful clinical benefit in most adult patients with R/R KMT2A-r leukemia. Notably, one patient who attained CR experienced rapid disease relapse upon discontinuation of therapy, suggesting that sustained DOT1L inhibition may be necessary to maintain remission—an idea that needs further investigation [46]. Another clinical study (NCT03724084) is evaluating the efficacy of Pinometostat combined with conventional chemotherapy in adult patients newly diagnosed with KMT2A-r AML. In these trials, Pinometostat induced temporary reductions in peripheral or bone marrow leukemic blasts in 7/18 patients, though these reductions did not meet formal criteria for an objective response [47]. Unfortunately, Pinometostat demonstrated limited efficacy in similar clinical trials for treating pediatric KMT2A-r leukemia (NCT02141828). A recent preclinical study indicated that Pinometostat enhances the sensitivity of pediatric AML cells to Sorafenib, a multi-kinase inhibitor, regardless of KMT2A-r status, suggesting that combination therapy with Pinometostat may still hold value in pediatric leukemia [48].

**Table 3:** DOT1L inhibitor (Pinometostat) in clinical trials

Agent	Disease	Phase	Enrollment	NCT.gov identifier	Status
**DOT1L inhibitor, Pinometostat (EPZ-5676)**					
Pinometostat in combination with Standard Induction Chemotherapy	Newly Diagnosed AML with KMT2A-r	Phase 1b/2	6	NCT03724084	Terminated
Pinometostat and Azacitidine	Patients With R/R, or Newly Diagnosed AML with KMT2A-r	Phase 1/2	1	NCT03701295	Completed
EPZ-5676	Pediatric Patients with R/​R Leukemias with KMT2A-r	Phase 1	18	NCT02141828	Completed
EPZ-5676	Adult Patients with R/R Leukemias with KMT2A-r	Phase 1	51	NCT01684150	Completed

### Menin

Menin, a nuclear protein primarily recognized for its tumor suppressor function, is encoded by the multiple endocrine neoplasia type 1 (MEN1) gene, which contains 10 exons and is located on chromosome 11q13. Menin regulates gene transcription, cellular growth, hematopoiesis, and myeloid proliferation. Menin has a unique role in interacting with histone-modifying proteins, such as KMT2A, which are critical for epigenetic regulation. It binds to DNA through nuclear localization sequences in its C-terminal region, regulating gene expression. Menin functions as a bridge between transcription factors and epigenetic effectors [49]. Several malignancies, including MEN1 invoving mutated Menin, and specific acute leukemias with KMT2A rearrangement, are significantly associated with Menin. Structural analysis of Menin reveals high conservation across species. Menin possesses a central cavity that serves as the binding site for protein-protein interactions (PPI). Menin binds to the N-terminal region of KMT2A, which is highly conserved across all KMT2A fusion proteins. The N-terminal fragment of KMT2A contains two short motifs, Menin binding motif 1 (MBM1) and Menin binding motif 2 (MBM2), which mediate the Menin-KMT2A interaction. The MBM1 binding site on Menin is well-exhibits higher affinity for Menin compared to MBM2. MBM1 and MBM2 compete for Menin proximal loci binding [50]. The interaction between KMT2A fusion proteins and Menin is crucial for the development of KMT2A-r leukemia, as Menin is a necessary cofactor for KMT2A binding to HOX gene promoters (Fig. 2). KMT2A fusion proteins translocate to the nucleus upon binding with Menin. Nuclear localization induces aberrant transcription of HOXA and other genes, which is essential for the development of KMT2A-r AML.

### Menin inhibitors

Multiple in vivo and in vitro studies have demonstrated that the Menin-KMT2A interaction can be effectively disrupted by targeting the MBM1-Menin binding. As a result, the MBM1-Menin interaction emerged as the most promising target for the developing Menin inhibitors composed of peptide corresponding to the MBM1 fragment [51, 52]. Inhibiting the binding between the Menin protein and KMT2A complex effectively blocks downstream signaling and promotes the differentiation of immature leukemic cells (Fig. 2). In 2005, Yokoyama et al. illustrated pathogenetic mechanism of the interaction between KMT2A and Menin [53]. A decade later, Borkin et al. developed the first Menin inhibitors, MI-463 and MI-503. These inhibitors were successfully tested in AML mouse models with KMT2A-rs, demonstrating a survival advantage without affecting normal hematopoiesis [54]. These findings were further validated in patient-derived xenograft models treated with two small molecules of the same class, VTP50469 and MI-3454, which exhibited a targeted mechanism of action, potent antileukemic activity, and extended survival without causing toxicity [55, 56].

Currently, clinical trials are evaluating seven oral Menin inhibitors, primarily as monotherapy for R/R acute leukemias. The inhibitors under evaluation include SNDX-5613 (Revumenib), KO-539 (Ziftomenib), DSP-5336, DS-1594, BN104, BMF-219, and JNJ-75,276,617 (Bleximenib). Table 4 summarizes all clinical trials evaluating Menin Inhibitors, either as monotherapy or in combination with other agents, in hematology malignancies.

**Table 4:** Clinical trials of Menin inhibitors

Agent	Disease	Phase	Enrollment	NCT.gov identifier	Status
**Revumenib (SNDX-5613)**					
Revumenib with Azacitidine + Venetoclax	Patients with NPM1-mutated or KMT2A-rearranged AML	Phase 3	415	NCT06652438	Not yet recruiting
Revumenib	Patients with KMT2A-Rearranged or NPM1-Mutated Acute Leukemia After AHSCT	Phase 1	27	NCT06575296	Not yet recruiting
Revumenib With 7 + 3 + Midostaurin	AML	Phase 1	22	NCT06313437	Not yet recruiting
Revumenib and Venetoclax	AML MRD positive	Phase 2	8	NCT06284486	Recruiting
Revumenib	Leukemia Associated with Upregulation of HOX Genes	Phase 2	15	NCT06229912	Recruiting
Revumenib and Gilteritinib	R/R FLT3-Mutated AML and Concurrent MLL-Rearrangement or NPM1 Mutation	Phase 1	30	NCT06222580	Recruiting
Revumenib, Azacitidine, and Venetoclax	in Pediatric and Young Adult Patients With R/R AML	Phase 1	24	NCT06177067	Recruiting
Revumenib + Daunorubicin and Cytarabine	Newly Diagnosed AML Patients with Changes in NPM1 or MLL/​KMT2A Gene	Phase 1	28	NCT05886049	Recruiting
Revumenib in Combination with Chemotherapy	Patients Diagnosed with R/R Leukemia	Phase 2	78	NCT05761171	Recruiting
Radiolabeled Revumenib	Adults with Acute Leukemia	Phase 1	8	NCT05406817	Recruiting
Revumenib in Combination with Chemotherapy	Participants With R/R Acute Leukemia	Phase 1	30	NCT05326516	Completed
Revumenib	R/R Leukemias Including Those with an MLL/KMT2A Gene Rearrangement or NPM1 Mutation	Phase 1/2	413	NCT04065399 (AUGMENT-101)	Recruiting
Ziftomenib (KO-539)					
Ziftomenib With Venetoclax and Gemtuzumab	Pediatric Patients With AML	Phase 1	22	NCT06448013	Not yet recruiting
Ziftomenib	Maintenance Post Allo-HCT in AML with NPM1m and KMT2A-r	Phase 1	22	NCT06440135	Recruiting
Ziftomenib, Venetoclax and Azacitidine	in Pediatric R/R Acute Leukemias	Phase 1	22	NCT06397027	Not yet recruiting
Ziftomenib in Combination with Chemotherapy	Children with R/R Acute Leukemia	Phase 1	20	NCT06376162	Not yet recruiting
Safety and Tolerability of Ziftomenib Combinations	KMT2A-r or NPM1-m R/R AML	Phase 1	171 e	NCT06001788	Recruiting
Ziftomenib with Venetoclax/​Azacitidine, Venetoclax, or 7 + 3	Patients with AML	Phase 1	212	NCT05735184	Recruiting
Ziftomenib	Patients With R/R AML	Phase 1/2	199	NCT04067336	Recruiting
DSP-5336					
DSP-5336	R/R AML/ ALL with or Without KMT2A-r or NPM1-m	Phase 1/2	70	NCT04988555	Recruiting
DS-1594b					
DS-1594bWith or Without Azacitidine, Venetoclax, or Mini-HCVD	R/R AML or ALL	Phase 1/2	17	NCT04752163	Completed
Bleximenib (JNJ-75276617)					
Bleximenib in Combination with Conventional Chemotherapy	Pediatric and Young Adult Participants with Relapsed/Refractory Acute Leukemias	Phase 1	0	NCT05521087	Withdrawn
Bleximenib in Combination with Directed Therapies	Patients with AML	Phase 1	200	NCT05453903	Recruiting
Bleximenib	patients With Acute Leukemia	Phase 1/2	400	NCT04811560	Recruiting
BMF-219					
BMF-219	Adult Patients With AML, ALL (With KMT2A/MLL1r, NPM1 Mutations), DLBCL, MM, and CLL/SLL	Phase 1	177	NCT05153330	Recruiting
BN104					
BN104	Acute Leukemia	Phase 1/2	90	NCT06052813	Recruiting

#### Revumenib (SNDX-5613)

Revumenib (commercial name Revuforj), an oral Menin inhibitor, was approved by the U.S. Food and Drug Administration (FDA) in December 2024 for the treatment of R/R acute leukemia with KMT2A translocations in adult and pediatric patients aged one year and older [57]. Revumenib (SNDX-5613) showed promise as a stand-alone treatment in the phase I/II study of patients with R/R AML who had KMT2A-r or NPM1 mutations (NCT04065399) [10]. A total of 68 highly pretreated individuals were included, comprising 56 patients (82%) with R/R AML, 11 patients (16%) with ALL, and one patient (2%) with NPM1 mutant AML. Forty-six patients (68%) had KMT2A-r, 14 (21%) had mutated NPM1 and eight (12%) had neither KMT2Ar nor NPM1 mutations. In total, the rate of CR/CRh was 30% in 18 of 60 with KMT2A-r or NPM1 mutation. The overall response rate (ORR) was 53% in 32 of 60 evaluable patients. The median time to CR/CRh was 1.9 months (range, 0.9–4.9). Fourteen patients (78%) achieved MRD negativity, confirmed by multiparameter flow cytometry. Eleven patients (16%) showed grade 3 or 4 treatment-related adverse event (TRAE). These findings prompted the ongoing Phase I/II AUGMENT-101 trial in the same patient population. In this study, twelve people who achieved a CR, morphological leukemia-free state (MLFS), or partial remission (PR) underwent stem cell transplantation (SCT) [58]. Patients discontinued Revumenib before SCT conditioning and resumed it 59–180 days post-SCT upon achieving CR. To date, 8/12 patients (66%) have resumed Revumenib, and 4/12 patients (33%) have discontinued it because of progressive disease or cytopenias. The median duration of response was 9.1 months, with a median follow-up of 11.9 months following the attainment of CR. CR was maintained in six patients (50%) following SCT and maintenance therapy with Revumenib [59]. In all, five patients (42%) sustained MRD-negative remissions [60]. A Phase I open-label trial (NCT05406817) is currently enrolling participants to evaluate the absorption, metabolism, and excretion (AME) of orally administered carbon-14 ([14 C]) SNDX-5613 in patients with R/R acute leukemia.

#### Ziftomenib (KO-539)

Another current phase I/II clinical trial, the KOMET-001 trial (NCT04067336), is investigating ziftomenib (KO-539) in patients with R/R AML with KMT2A-r or NPM1 mutations. In the 2022 update, thirty patients participated in the dose-finding phase, indicating that a 600 mg dose was both feasible and effective, with an ORR of 42%, a CR or CR with partial hematologic recovery (CRh) rate of 25%, and a CR with incomplete hematologic recovery (CRi) rate of 33.3%. As of the April 2023 data cutoff, 35% of patients with NPM1mutations AML who received the recommended phase 2 dose (RP2D) of 600 mg attained CR [61]. Ziftomenib is currently being investigated, as well as their synergistic effects with other targeted agents [62].

#### DSP-5336

An ongoing phase I/II trial of DSP-5336 is evaluating patients with R/R acute leukemia (NCT04988555) [63]. Patients with R/R AML, ALL, or acute leukemia of indeterminate origin were eligible, regardless of prior therapies, particularly those with KMT2A-r and NPM1mutations. No DLT has been observed. Most adverse events were classified as Grade 1 or 2. A potential grade 4 differentiation syndrome was noted. Among the six patients enrolled with R/R AML and KMT2A-r, one achieved CR with incomplete blood count recovery (CRi), another reached a morphologic leukemia-free state, and a third exhibited stable disease, characterized by the elimination of peripheral blasts, recovery of peripheral blood counts, and a decrease in bone marrow blasts from 85 to 31%. Among the four patients with NPM1 mutations, two exhibited disease stability, with complete eradication of peripheral blasts and a reduction of 66% and 83% in bone marrow blasts, respectively [63].

#### DS-1594b

A recent study has demonstrated the in vitro efficacy of DS-1594b as monotherapy or in combination with Venetoclax in KMT2A-r and NPM1-mutation cell lines, as well as in primary leukemia samples. Initial data indicated that DS-1594b did not induce apoptosis as a monotherapy in any tested AML cell line, suggesting that differentiation is a crucial mechanism of Menin inhibition [64, 65]. A phase I/II trial (NCT04752163) investigated the efficacy of DS-1594b as a monotherapy and in combination with Azacitidine and Venetoclax or mini-HCVD for the treatment of patients with R/R AML and ALL. Additionally, both the hydrochloride and succinate forms of DS-1594a demonstrated strong anti-leukemic effects in preclinical studies using AML/ALL models, which supports the next step of testing oral doses in clinical trials (NCT04752163). In KMT2A-rearranged AML cells, DS-1594a promoted differentiation and reduced the serial colony-forming potential, indicative of its capacity to target leukemia-initiating cells. Notably, both variants of DS-1594a were highly effective in eradicating tumors and enhancing survival in MOLM-13 cells and xenograft models of KMT2A-r or NPM1-m acute leukemia derived from patients [66].

#### JNJ-75,276,617

JNJ-75,276,617 is an effective, selective inhibitor targeting the Menin-KMT2A interaction, demonstrating efficacy in leukemia cell lines and patient samples with KMT2A-rs or NPM1 mutations. As of the 2023 update, 58 patients received JNJ-75,276,617; 56 patients had R/R AML, and 2 patients had R/R ALL. A KMT2A or NPM1 mutation was identified in 33 (57%) and 25 (43%) patients, respectively. Grade 3 or higher adverse events occurred in 17 patients (29%), including neutropenia (10%), anemia (7%), and thrombocytopenia (7%). Five patients (9%) had DLTs, including differentiation syndrome (3%). Disease burden diminished in 63% (26/41) of patients, with 39% (16 patients) showing a reduction of over 50% in bone marrow blasts. At the highest dose (90 mg twice a day; 8 patients), the overall response rate was 50% (4 patients), including 2 patients with NPM1 mutation and 2 with KMT2A-r, resulting in complete response (1 patient), complete response with partial hematologic recovery (1 patient), and clinical response (2 patients). In patients taking 45 mg twice a day (*n* = 20), the ORR was 40% (8 patients), which included 5 patients with NPM1 mutation and 3 with KMT2A-r, achieving CR (3 patients), CRh (1 patient), Cri (3 patients), and PR (1 patient). The average time to response (PR or better) was 1.81 months, and time to CR/CRh/Cri was 1.77 months. Twelve responses were noted, including one instance of MRD-negative full remission. Initial pharmacodynamic data indicated a reduced expression of Menin–KMT2A target genes and alterations in differentiation-associated genes in individuals who exhibited a response [63].

#### BMF-219

A phase I clinical trial involving dose escalation and dose expansion (NCT05153330) of BMF-219, an oral covalent Menin inhibitor, is currently underway through 4 cohorts for adult patients with R/R acute leukemia with KMT2A-r or NPM1 mutation (Cohort 1), diffuse large B cell lymphoma (DLBCL) (Cohort 2), multiple myeloma (MM) (Cohort 3), and chronic lymphocytic leukemia (CLL)/small lymphocytic lymphoma (SLL) (Cohort 4). As of July 2023, 26 patients with R/R acute leukemia (24 AML and 2 ALL) were enrolled. The median number of prior therapy lines was 4 (range: 1–8), with 11 patients having a history of SCT. BMF-219 was administered daily over a continuous 28-day cycle until disease progression or treatment intolerance occurred. The BMF-219 has been generally well-tolerated, with no DLTs observed and no discontinuations due to treatment-related toxicities. Vomiting (13%) and differentiation syndrome (13%) were among the most prevalent adverse effects. So far, the first results have been reported for two patients with AL from Cohort 1—one has an NUP98-NSD1 fusion (Patient A) and the other has an NPM1 mutation (Patient B). At cycle 1, day 27 (C1D27), Patient A’s BM blasts decreased from 13% at baseline to 6%, and the patient achieved CR with 0% blasts at C2D28. For Patient B, BM blasts were reduced from 52% at baseline to 34% at C1D28, and the patient achieved a CRi with 3% blasts at C2D28. Also, the initial findings indicated preliminary evidence of therapeutic efficacy across several genetic subgroups, such as patients with KMT2A-r, KMT2A-PTD, NPM1, and WT for KMT2A and NPM1 [67].

### Assessing the response to Menin inhibitors

A potential biomarker for assessing the response to Menin inhibitors is the analysis of HOX (A and/or B) gene expression alongside levels of its cofactor, MEIS1. The interaction between Menin and the KTM2A protein is essential for the leukemic transcriptional pathway in AML, deriven by increased HOX/MEIS1 gene expression [51]. Menin inhibitors disrupt the Menin–KMT2A interaction, resulting in the downregulation of HOX and MEIS1 transcription and inhibition of leukemogenesis. Recent studies validate HOXA9/MEIS1 transcript levels as dynamic biomarkers for Menin inhibitor response, with reductions ≥ 50% correlating with prolonged survival [68].

### Targeting wild-type KMT2A

Targeting the wild-type (WT) KMT2A protein in KMT2A-r leukemia represents a promising therapeutic approach, garnering increasing interest in current research. In KMT2A-r, the WT KMT2A protein is essential for maintaining the abnormal gene expression patterns and promotes the recruitment of essential histone modifications, including H3K4 and H3K79 methylation, which are vital for sustaining the leukemic transcriptional pathway [69]. Consequently, the interaction between WT KMT2A protein and KMT2A fusion proteins has generated interest in therapies specifically targeting the WT KMT2A protein. Various KMT2A-associated malignancies, especially KMT2A-PTD and KMT2A-a leukemias, which exhibit unfavorable prognoses and require improved therapeutic options, may benefit from WT KMT2A inhibitors. Early efforts to inhibit WT KMT2A function have concentrated on disrupting its interaction with WDR5, a protein essential for KMT2A’s methyltransferase activity [70]. Inhibition of WT KMT2A methyltransferase activity specifically impairs the proliferation of KMT2A-r cells by inducing cell-cycle arrest, increasing apoptosis, and initiating myeloid differentiation. This inhibition does not induce general toxicity in normal bone marrow cells or non-MLL cells [70]. Recent preclinical studies have demonstrated that peptidomimetic and small-molecule inhibitors disrupting the KMT2A/WDR5 interaction could serve as potential therapies for KMT2A-r leukemia [71]. Furthermore, innovative methods such as PROTAC technology, which selectively degrades core components of the KMT2A complex (WDR5 and ASH2L), have shown promise in changing the KMT2A-mediated epigenetic landscape and gene expression [72]. The investigation of these novel modalities has the potential to facilitate the development of more effective treatments for patients with KMT2A-related leukemias and other cancers that are influenced by KMT2A aberrations.

## Future direction and challenges

One of the primary challenges in managing KMT2A-rearranged AML is the marked molecular heterogeneity exhibited by these leukemias, which complicates both diagnosis and risk stratification [12]. This heterogeneity stems from the diversity of fusion partners associated with KMT2A-r, each imparting distinct biological and clinical characteristics that can influence disease progression and response to therapy [73]. The aggressive clinical behavior observed in KMT2A-rearranged AML, characterized by high relapse rates and poor overall survival, further underscores the urgent need for improved therapeutic strategies [74]. Standard chemotherapeutic regimens have proven insufficient in achieving durable remissions in this subgroup of AML patients, largely due to intrinsic resistance mechanisms that remain poorly understood [3, 75]. Moreover, the current lack of reliable predictive biomarkers hampers the ability to tailor treatment approaches effectively, as clinicians are often unable to determine which patients will benefit from specific therapies [76]. In addition to these challenges, the interplay between aberrant epigenetic modifications and dysregulated transcriptional programs in KMT2A-rearranged AML further complicates the disease biology, limiting the scope of conventional treatment modalities [12].

Looking forward, future research must focus on elucidating the precise molecular mechanisms driving KMT2A-mediated leukemogenesis, as a deeper understanding in this area could reveal novel therapeutic targets [73]. A promising avenue is the development of targeted therapies, such as Menin inhibitors, which have shown encouraging preclinical efficacy by disrupting the critical interaction between Menin and KMT2A fusion proteins [74]. These targeted agents are anticipated to shift the current treatment paradigm by directly interfering with the oncogenic transcriptional machinery rather than relying solely on cytotoxic approaches [75]. Despite promising outcomes from early-phase clinical trials (Phases I/II) investigating Menin inhibitors for KMT2A-rearranged AML and other AML subtypes, these agents have not been incorporated into standard clinical practice. A multitude of significant elements contribute to this. First, Menin inhibitors must be validated in larger, randomized Phase III studies to establish their superiority—or at least equivalence—compared to current standard-of-care regimens, despite the fact that initial trials have shown promising therapeutic activity and favorable safety profiles. Second, there is a significant lack of long-term data on the potential for late-emerging toxicities, the durability of clinical responses, and the overall survival benefits. For example, the AUGMENT-101 trial showed a median overall survival of 9 months and a median duration of CR or CRh of 6.4 months [77]. A similar median follow-up of 9.3 months was reported by the SAVE trial, which examined a combination therapy that included revumenib; the median duration of CR/CRh had not yet been reached [78].Third, recent research suggests that the sustained efficacy of Menin inhibition may be compromised by the likelihood of resistance developing over time. Studies have identified specific MEN1 mutations that confer resistance to Menin inhibitors, emphasizing the difficulties in sustaining long-term efficacy [79–81]. Fourth, the criteria for optimal patient selection and the identification of predictive biomarkers are still in the process of evolving. Despite the fact that KMT2A-rs and NPM1 mutations are recognized molecular targets, the impact of other genetic and clinical factors on the response to treatment is not yet completely understand [82, 83].

In parallel, emerging CD123-directed CAR T-cell therapies, such as MB-102 (NCT04109482), have shown preclinical efficacy in KMT2A-r AML, offering a novel combinatorial approach with Menin inhibitors [76, 84, 85]. Advances in genomic profiling and high-throughput screening are expected to facilitate the identification of new biomarkers that can predict therapeutic response and monitor MRD, thus allowing for more precise patient stratification [12]. Furthermore, integrating these molecular insights into clinical trial designs will be essential for evaluating the efficacy of combination regimens that incorporate both targeted therapies and immunomodulatory agents [73]. Such combination strategies could not only enhance anti-leukemic activity but also mitigate the emergence of resistance, which remains a significant hurdle in the treatment of KMT2A-rearranged AML [74, 84]. Additionally, understanding the mechanisms underlying treatment resistance will be critical in developing next-generation inhibitors that can circumvent or delay resistance pathways, thereby improving long-term outcomes [75]. Finally, a multidisciplinary approach that combines insights from molecular biology, immunology, and clinical oncology is imperative to translate these scientific advances into meaningful clinical benefits for patients with this challenging leukemia subtype [76]. Collectively, addressing these challenges through continued research and innovation holds the promise of revolutionizing the management of KMT2A-rearranged AML, ultimately leading to more personalized and effective therapeutic strategies that can significantly improve patient survival and quality of life [12, 86].

## Conclusion

KMT2A-r in AML represent a paradigm of epigenomic dysregulation, driving aggressive leukemogenesis through the hijacking of transcriptional machinery and sustained activation of proto-oncogenic pathways such as HOXA/MEIS1. Advances in molecular diagnostics, including NGS and machine learning-driven biomarker discovery, have enabled earlier and more precise identification of high-risk cohorts. Therapeutic innovation has transitioned from conventional chemotherapy to targeted epigenetic modulation, with Menin inhibitors (e.g., Revumenib, Ziftomenib) and DOT1L inhibitors (e.g., Pinometostat) demonstrating remarkable clinical potential. Building on the first FDA‑approved Menin inhibitor (revumenib), this review delineates emerging PROTAC‑ and WDR5‑targeted approaches, machine‑learning–predicted biomarkers, and nascent immunotherapies, offering a comprehensive roadmap for next‑generation, precision‑guided management of KMT2A‑rearranged AML. Looking forward, integrating molecular profiling, novel combinatorial regimens, and post-transplant maintenance strategies may further enhance patient outcomes. While unresolved questions regarding clonal heterogeneity and resistance mechanisms necessitate further investigation, the convergence of precision diagnostics and epigenetically targeted therapies heralds a transformative era in KMT2A-r AML management. By integrating molecular insights with clinical innovation, these advances are poised to redefine prognostic paradigms and ultimately improve survival for patients with this historically intractable leukemia.

## Data Availability

No datasets were generated or analysed during the current study.
